# An RNA-Seq Strategy to Detect the Complete Coding and Non-Coding Transcriptome Including Full-Length Imprinted Macro ncRNAs

**DOI:** 10.1371/journal.pone.0027288

**Published:** 2011-11-10

**Authors:** Ru Huang, Markus Jaritz, Philipp Guenzl, Irena Vlatkovic, Andreas Sommer, Ido M. Tamir, Hendrik Marks, Thorsten Klampfl, Robert Kralovics, Hendrik G. Stunnenberg, Denise P. Barlow, Florian M. Pauler

**Affiliations:** 1 CeMM Research Center for Molecular Medicine of the Austrian Academy of Sciences, Vienna, Austria; 2 Research Institute of Molecular Pathology, Vienna, Austria; 3 Department of Molecular Biology, Faculty of Science, Nijmegen Center for Molecular Life Sciences (NCMLS), Radboud University Nijmegen, Nijmegen, The Netherlands; Istituto Dermopatico dell'Immacolata, Italy

## Abstract

Imprinted macro non-protein-coding (nc) RNAs are cis-repressor transcripts that silence multiple genes in at least three imprinted gene clusters in the mouse genome. Similar macro or long ncRNAs are abundant in the mammalian genome. Here we present the full coding and non-coding transcriptome of two mouse tissues: differentiated ES cells and fetal head using an optimized RNA-Seq strategy. The data produced is highly reproducible in different sequencing locations and is able to detect the full length of imprinted macro ncRNAs such as *Airn* and *Kcnq1ot1*, whose length ranges between 80–118 kb. Transcripts show a more uniform read coverage when RNA is fragmented with RNA hydrolysis compared with cDNA fragmentation by shearing. Irrespective of the fragmentation method, all coding and non-coding transcripts longer than 8 kb show a gradual loss of sequencing tags towards the 3′ end. Comparisons to published RNA-Seq datasets show that the strategy presented here is more efficient in detecting known functional imprinted macro ncRNAs and also indicate that standardization of RNA preparation protocols would increase the comparability of the transcriptome between different RNA-Seq datasets.

## Introduction

The study of whole transcriptomes, which detects all RNAs expressed in one cell population, provides insights into mechanisms regulating gene expression in development and disease. The first large-scale mammalian transcriptome studies based on genome tiling array hybridization or cDNA sequencing, unexpectedly revealed that most RNAs excluding structural tRNAs, rRNAs and spliceosome RNAs were non-protein-coding (nc) RNAs [1], [2], [3]. This ncRNA population, which is considered to play a pivotal role in regulating gene expression, is currently classified according to size [4]. Small ncRNAs include siRNAs, miRNAs and piRNAs that are less than 30 bp and are involved in transcriptional and post-transcriptional gene silencing, and also snoRNAs that are less than 200 bp and primarily guide rRNA chemical modifications. These small ncRNAs can be processed from mRNA introns or from independent long or macro precursor ncRNAs [5], [6], [7]. Macro ncRNAs that are currently defined as transcripts greater than 200 bp but can exceed 100 kb in length, function without being processed into small ncRNAs. Well-known examples of functional macro ncRNAs in mammals are imprinted macro ncRNAs of which *Airn*, *Kcnq1ot1* and *Nespas* have been shown to repress 1–10 genes *in cis* [8], [9], [10].

The development of Next Generation Sequencing applied to RNA (referred to as RNA-Seq) provides an accurate, unbiased and quantifiable transcriptome [11], [12]. Different technologies such as the Illumina/Solexa and ABI SOLiD platforms use cDNA fragment clusters to produce short sequencing tags without vector subcloning [13]. Transcriptome analysis by RNA-Seq is a multi-step protocol that can be divided in three main parts. The first part termed ‘template preparation’ includes the isolation of total RNA from cells or tissues and the preparation of fragmented cDNA. The second part usually referred to as ‘sample preparation’ includes all steps to produce a library from the fragmented cDNA that is used by the sequencing platform to generate millions of short sequence reads or tags. In the last part, bioinformatic procedures convert the sequence tags into an interpretable format by aligning them to a reference genome and quantifying expression levels of annotated genes [14].

The ease and cost effectiveness of RNA-Seq has led to a growing amount of publications that share their transcriptome studies with the scientific community [11], [15]. This raises important issues on the need to optimize RNA-Seq pipelines to ensure they represent the complete coding and non-coding transcriptome as well as to ensure reproducibility and comparability of transcriptomes generated at different sequencing locations. It is becoming clear now that differences in template preparation protocols create biases in the detection of gene expression and several bioinformatic applications have been developed to compensate for this bias [16], [17]. However compared to the rapid development of bioinformatic analysis tools, little progress has been made in standardizing template preparation protocols in a similar manner as proposed for microarray expression platforms [18]. Most current knowledge of the strengths and weaknesses of RNA-Seq is based on studies using polyA enriched RNA as a starting material [19], [20]. Currently very little information is available on the ability of RNA-Seq to detect the full ncRNA transcriptome, and information is particular lacking about the detection of imprinted macro ncRNAs.

The majority of cellular RNA is composed of ribosomal RNAs (rRNAs). As sequencing this part of the transcriptome is usually not the main research focus, the RNA sample is prepared either by selection of polyadenylated (polyA) RNAs or by selective removal of rRNAs (ribo-depletion). RNA-Seq using polyA selected RNA detects protein-coding mRNAs at a depth sufficient to determine tissue-specific gene expression differences and can also detect at least a subset of ncRNAs [19], [20]. However, it has also been shown that the template fragmentation step induces important differences in the gene coverage by sequence tags and that the 3′UTRs are under-represented in polyA RNA-Seq [12], [20], [21]. The effect of ribosomal RNA depletion on RNA-Seq (ribo-depleted RNA-Seq) and the resulting transcriptome has been less studied. It is generally assumed that ribo-depleted RNA-Seq may perform better than polyA RNA-Seq, as it will detect ncRNAs lacking a polyA tail and those that are very long and likely to be broken into fragments lacking a polyA tail. A recent study investigated the use of ribo-depleted RNA-Seq for detecting the protein-coding and non-coding transcriptome [22], but several important issues have not yet been addressed. These include: (i) the influence of template fragmentation on gene expression and gene coverage, (ii) the reliability of expression levels determined by ribo-depleted RNA-Seq, (iii) the reproducibility of ribo-depleted RNA-Seq when using different sequencing machines and ribosomal RNA depletion methods, and (iv) the advantages of ribo-depleted RNA-Seq over polyA enriched RNA-Seq in the detection of macro ncRNAs.

In order to address these issues we modified an established protocol for RNA-Seq using the Illumina/Solexa platform [20] and used it to analyse the non-ribosomal transcriptome of two distinct mouse tissues by RNA-Seq. In brief this protocol includes column-free RNA preparation, ribosomal RNA depletion, RNA-hydrolysis and ds-cDNA synthesis. The resulting ds-cDNA is compatible with the needs of the major NGS platforms used to date [13]. We first show that ribo-depleted RNA-Seq is highly reproducible between different sequencing locations, even when using two different ribosomal RNA depletion strategies. Secondly, we show that template fragmentation by RNA-hydrolysis produces more homogenous gene coverage than cDNA shearing, and that both fragmentation methods lead to under-representation of 5′ and 3′ UTRs. Notably, by comparing the protein-coding transcriptomes obtained here to published RNA-Seq datasets from similar tissues, we show that the use of similar template preparation protocols is critical for obtaining a comparable transcriptome. Lastly, we show that RNA populations prepared by ribo-depletion allow RNA-Seq to reliably detect both the non-coding and protein-coding transcriptome, and also to identify biologically relevant gene expression differences in both these RNA types between the two analysed mouse tissues. Importantly our template preparation protocol allowed the detection of well-studied imprinted macro ncRNAs that failed to be identified in comparable polyA and ribo-depleted RNA-Seq datasets that used different template preparation protocols.

## Results and Discussion

RNA for sequencing was obtained from retinoic acid differentiated mouse CCE embryonic stem cells and 14.5 dpc mouse fetal head (FH) and subject to ribosomal RNA depletion using either the RiboMinus Kit (Invitrogen) or the Ribo-Zero Kit (Epicentre, see Materials and Methods). FH is a complex combination of multiple tissues but primarily contains fetal brain whereas differentiated CCE cells resemble an extra-embryonic epithelial cell type called primitive endoderm [23]. The number of tags and the GEO accession number for each sequenced lane are shown in Table S1.

### RNA-Seq at different locations produces highly similar results

**Figure 1:**
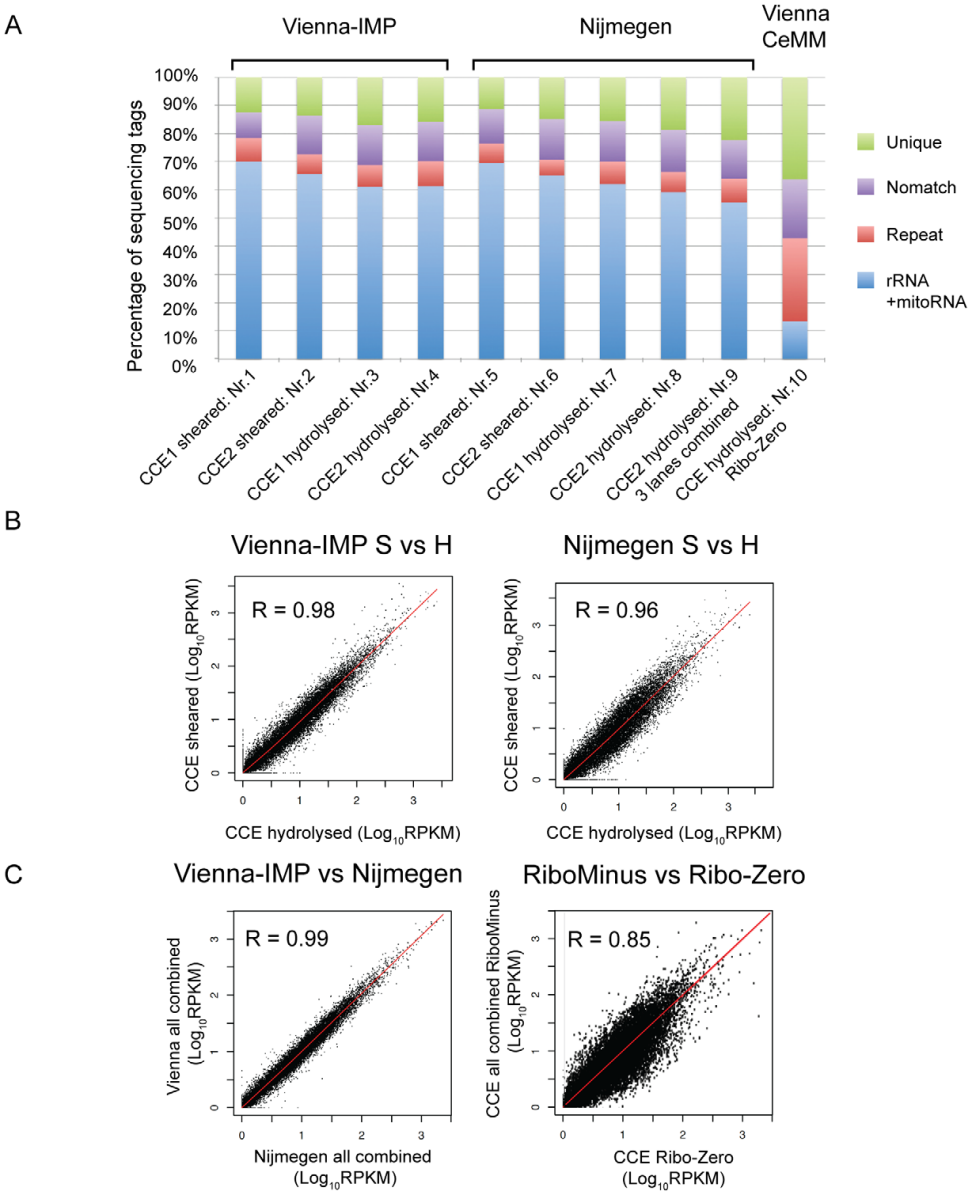
Optimisation and reproducibility of ribo-depleted RNA-Seq. (**A**) Distribution of different sequence tag types from RNA prepared from CCE differentiated ES cells subject to ribosomal RNA depletion using either the RiboMinus or the Ribo-Zero Kit and fragmented either by RNA-hydrolysis or by cDNA-shearing. Sequencing was performed in two different sequencing locations (Vienna-IMP, Nijmegen, RiboMinus) or in one sequencing location (Vienna-CeMM, Ribo-Zero). The percentage of tags in each category is shown for two technical sequencing replicates (CCE1, CCE2) of material prepared by RiboMinus and cDNA-shearing (sheared, lanes nr. 1,2,5,6) or RiboMinus and RNA-hydrolysis (hydrolysed, lanes nr. 3,4,7,8), for the combination of three technical sequencing replicates of RiboMinus and RNA-hydrolysis (lane nr. 9) and for one sequencing of Ribo-Zero and RNA-hydrolysis (lane nr. 10). green: unique tags matching only once in the genome; blue: rRNA+mitoRNA tags matching to ribosomal (RiboMinus and Ribo-Zero) or mitochondrial (RiboMinus) RNAs; red: repeat tags matching more than once in the genome; purple: nomatch tags do not match to the genome. (**B**) Scatter plots comparing the RPKM (Reads Per Kilobase of exon model per Million of reads) transcription levels of RefSeq protein-coding genes between combined tags from RiboMinus and RNA-hydrolysis (H) and RiboMinus and cDNA-shearing (S) from CCE within the same location: Vienna-IMP (left) and Nijmegen (right). (**C**) Scatter plots as in B comparing RPKM transcript levels of all combined tags from the two sequencing locations (Vienna-IMP and Nijmegen, left) or between the combined RiboMinus data and the Ribo-Zero data (right). R: Pearson's correlation, note that a perfect correlation is R = 1.

For CCE cells, we prepared two technical replicates of one-round RiboMinus and for FH we prepared one or two rounds of RiboMinus (Table S1). To directly compare two fragmentation methods RNA-hydrolysis and cDNA-shearing, we applied both methods to the same RiboMinus samples. The same cDNAs were sequenced in two locations: Vienna-IMP (Research Institute of Molecular Pathology) and Nijmegen NCMLS (Nijmegen Centre for Molecular Life Sciences). We found that 46–73% (RiboMinus) of tags mapped to ribosomal / mitochondrial sequences (blue boxes in Figure 1A and Figure S1A). We then mapped the remaining sequence tags to the mouse genome (mm9). Overall we found 9–24% (RiboMinus) of tags mapped once in the genome (green boxes), 4–9% (RiboMinus) multiple times (red boxes) and 9–21% (RiboMinus) of tags did not match any dataset (purple boxes in Figure 1A and Figure S1A). As multiple RiboMinus datasets were available we analysed these in more detail. There was no significant difference detectable in any tag group between the two tissues, CCE and FH, when the sequencing was performed at the same location (Vienna-IMP or Nijmegen) using RNA-hydrolysis and at least two replicates per tissue (t-test, p>0.05). A similar comparison was not possible for cDNA-shearing as we only sequenced one FH sample at the two locations (Table S1). Importantly we found no significant differences in any tag group when comparing the data prepared in two different locations (Vienna-IMP and Nijmegen) from RNA-hydrolysis or cDNA-shearing irrespective of the tissue with at least three replicates per location (t-test, p>0.05), indicating high reproducibility of the technique. We did note a higher variability in the results for FH, however there was no trend for less ribosomal tags between one or two rounds of RiboMinus ribosomal RNA depletion (compare FH1 and FH2 Figure S1A). This finding confirms earlier reports that different locations produce similar proportions of sequence tags [24].

### RNA-hydrolysis and Ribo-Zero minimise ribosomal sequencing tags

We decided to expand this analysis to test if any significant difference was detectable between the different fragmentation methods. Therefore we compared all data from RNA-hydrolysis to all data from cDNA-shearing, irrespective of the tissue or location (Vienna-IMP and Nijmegen). This comparison shows more unique and nomatch as well as less ribosomal/mitochondrial tags were obtained from RNA-hydrolysis compared to cDNA-shearing (t-test, p<0.05). The number of repeat tags did not change significantly between the two fragmentation methods (t-test, p>0.1). This indicates that RNA-hydrolysis performs better than cDNA-shearing in producing more unique and less ribosomal RNA tags and is therefore the favourable template fragmentation method.

To optimise ribosomal RNA depletion we used an improved depletion method, Ribo-Zero, in combination with RNA-hydrolysis. For the RNA from both FH and CCE tissues, one Ribo-Zero treatment was performed, the RNA was hydrolysed and sequenced in Vienna-CeMM (Research Center for Molecular Medicine). This resulted in a more efficient removal of ribosomal RNAs as only 4–14% of all tags were of ribosomal origin. We also observed an increase in uniquely matching (36–54%) and multiple matching tags (28–30%) (blue, green, red boxes Figure 1A lane Nr.10 and Figure S1A lane Nr.8). Interestingly tags with nomatch in the genome did not change (14–21%, purple boxes Figure 1A Nr.10 and Figure S1A lane Nr.8). This analysis is, to our knowledge, the first one to use the combination of Ribo-Zero and RNA-hydrolysis on mouse tissues and we show this approach efficiently removes ribosomal RNAs and generates a high number of uniquely matching sequencing tags.

### Gene expression is reproducible between RNA depletion methods and sequencing locations

In the next step we analysed if the gene expression levels of protein-coding genes were comparable between the different fragmentation methods and locations. To obtain a comparable measure of gene expression we used RPKM (Reads Per Kilobase of exon model per Million mapped reads) values that are corrected for gene length and the total number of sequence tags. Although FPKM (Fragments Per Kilobase of exon model per Million mapped reads) values are starting to replace RPKM, the latter are widely used to estimate expression levels of protein-coding genes as well as ncRNAs [20], [21], [25], [26] and are useful to unravel technical bias in RNA-Seq protocols [15]. First we focused on RefSeq protein-coding genes in the RiboMinus datasets. A comparison of RNA-hydrolysis and cDNA-shearing showed that the RPKM values correlate well within the same tissue, sequenced in two different locations (Vienna-IMP and Nijmegen, Pearson correlation: R>0.9, Figure 1B and Figure S1B). Due to this high correlation, we pooled all tags from RNA-hydrolysis and cDNA-shearing for the two sequencing locations (Vienna-IMP and Nijmegen), recalculated the RPKM values and compared them to each other (Figure 1C left and Figure S1C left). Again we found a very good correlation for both CCE and FH (R>0.98). Finally we compared the RPKM values from the combined RiboMinus (Vienna-IMP and Nijmegen) to the Ribo-Zero datasets and found a high correlation for both tissues (R>0.8, Figure 1C right, Figure S1C right). This analysis shows that different ribosomal RNA depletion strategies and the sequencing at different locations produce comparable protein-coding gene expression levels.

### RNA-hydrolysis produces homogenous coverage of coding exons

**Figure 2:**
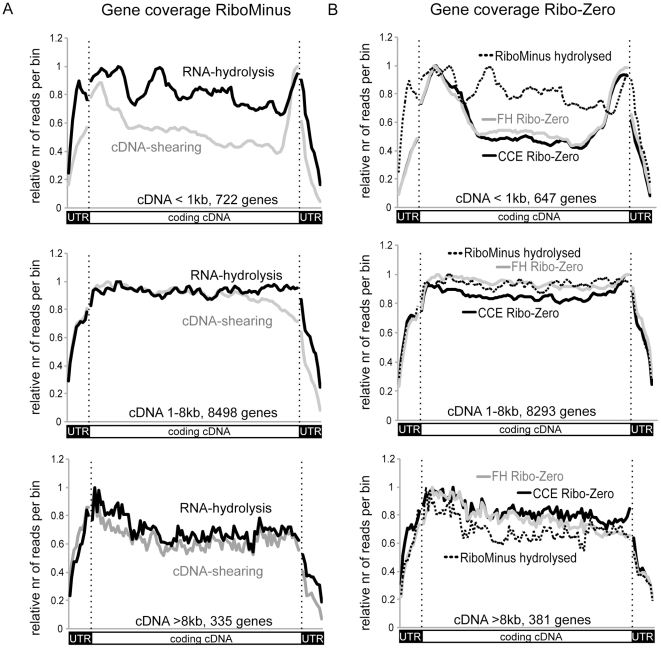
Tag coverage of genes differs between fragmentation methods and ribosomal RNA depletion methods. The coverage of genes with sequence tags is shown as the normalized number of tags at relative positions throughout the gene length. UTRs and coding exons were analysed separately and are plotted as 10 bins for 5′UTRs and 3′UTRs and 100bins for the coding exons (separated by vertical dotted line). (**A**) Comparison of the coverage in the RiboMinus dataset for the combined tags of CCE and FH from RNA-hydrolysis (black) and cDNA-shearing (grey). (**B**) Comparison of the coverage in the RNA-hydrolysis RiboMinus dataset (dotted line, same as in A) and in Ribo-Zero dataset plotted separately for CCE (black) and FH (grey). For all analyses the genes were separated into three groups according to their cDNA length (coding exons and 5′ and 3′ UTRs) as indicated.

A recent review reported that chemical breakdown of RNA (RNA-hydrolysis) followed by cDNA synthesis produced a more equal representation of genes with sequencing tags than physically breaking up the cDNA (cDNA-shearing). In particular, this review noted an extreme 3′ positive bias for protein-coding genes from cDNA sheared templates in polyA RNA-Seq [12]. As this review analysed RNA-hydrolysis data from mouse [20] and cDNA-shearing data from yeast [27] we decided to directly compare for the first time both fragmentation methods, using the same RNA, for differences in gene coverage. We combined the RiboMinus datasets from both CCE and FH as we found that gene coverage was similar between these tissues (data not shown, also demonstrated by [21]), and compared RNA-hydrolysis and cDNA-shearing. We analysed coding regions and untranslated regions (UTRs) of protein-coding mRNAs separately. Note that UTRs are found at both the 3′ and the 5′ end of protein-coding mRNAs and can contain introns [28]. We then separately plotted the relative tag coverage of genes that are reliably expressed in CCE and FH (RPKM>3, see below) for three cDNA size groups: small (<1 kb), medium (1–8 kb) and large (>8 kb) (Figure 2A top, middle, bottom). We compared RNA-hydrolysis (black line) and cDNA shearing (grey line) for all gene size classes (Figure 2A). In all cases both the 5′ and 3′ UTRs (black boxes Figure 2) showed a large loss of tag coverage compared to coding exons. The coverage of the coding exons differed amongst different size classes. Small genes showed an increased coverage of their 5′ and 3′ ends for cDNA-shearing that was less pronounced when using RNA-hydrolysis (Figure 2A top). For medium-sized genes both fragmentation methods produced similar coverage at the 5′ end, but cDNA shearing showed a loss of coverage towards the 3′ end whereas RNA-hydrolysis produced a homogenous coverage throughout coding exons (Figure 2A middle). Large genes showed a similar picture for both fragmentation methods with increased tag coverage at the 5′ end of coding exons (Figure 2A bottom). This analysis indicates that in RiboMinus RNA-Seq coding exons are more homogenously covered than UTRs and that RNA-hydrolysis produces a more homogenous tag distribution over coding exons than cDNA-shearing. The data also shows no extreme 3′ positive bias for cDNA-shearing compared to RNA-hydrolysis. It is possible that the published extreme 3′ positive bias found for cDNA-shearing was at least in part produced by the use of oligo-dT primers rather than by the fragmentation method itself, however, this point was not directly investigated in this study [12].

We next compared the gene coverage in the Ribo-Zero dataset to the RNA-hydrolysis RiboMinus dataset. We again observed a loss of sequence tags in both the 5′ and the 3′ UTRs. In comparison to the RiboMinus preparation, Ribo-Zero produces more tags in the 5′ and 3′ ends of small genes compared to the body of the genes (Figure 2B top), whereas the coverage of the other gene sizes was largely unchanged between the two ribosomal RNA depletion methods (Figure 2B middle and bottom). This indicates that with the exception of small genes both ribosomal RNA depletion methods produce similar gene coverage patterns. While it is not clear what causes the difference in coverage of small genes, this data clearly shows the influence of the template preparation protocol on the coverage of genes by sequence tags. It is therefore possible that the choice of the template preparation method helps to improve coverage biases that originate from sequence specific features that influence the alignment of sequence tags [29].

We observed an under-representation of both 5′ and 3′ ends of genes of three size classes in sequencing tags from both RNA-hydrolysis and cDNA-shearing templates and for both ribosomal RNA depletion methods. We could directly link this phenomenon to 5′ and 3′ UTRs by analysing them separately from the coding regions. A similar drop of tag coverage at gene ends compared to gene bodies was previously reported, but was not connected to UTRs [12], [22]. Interestingly a large scale study of different polyA RNA-Seq datasets found discordance between expression values calculated with and without 3′ UTRs, indicating a lower coverage specific to 3′ UTRs as we show here [21]. However an equally poor coverage of 5′UTRs has not been previously reported. Our findings are important for future analyses as they indicate that factors independent of the polyA enrichment or the ribosomal RNA depletion cause this loss in tag coverage. Independent of the cause, this finding has important implications for quantifying gene expression from RNA-Seq datasets. We show that the proposal to use 3′ ends to quantify gene expression [27], might lead to inaccurate results due to the low tag coverage of UTRs in mammals. We support the suggestion that the exclusion of 3′ UTRs would more accurately quantify expression [21] and expand this suggestion to the additional removal of 5′ UTRs.

### Gene expression by ribo-depleted RNA-Seq reliably detects protein-coding and non-coding RNAs

**Figure 3:**
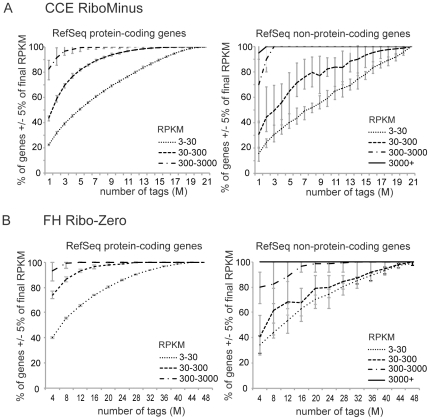
Ribo-depleted RNA-Seq reliably detects expression of known protein-coding and non-coding genes. (**A**) Saturation curves showing the percentage of RefSeq protein-coding genes (left) or RefSeq ncRNAs (right) with an RPKM+/−5% of the final RPKM for the combined CCE RiboMinus dataset (calculated at the maximum tag number) at different sequencing depths crated by randomly picking the indicated number of tags (M: million). The lines show three groups of genes with similar RPKM expression levels. Error bars indicate the minimum and maximum of ten random tag sets. If the curves reach a plateau before the final number of tags, this indicates that this gene group was sequenced exhaustively, as obtaining more tags does not change their RPKM. The large error bars originate from small gene numbers in the categories, where a small number of changed genes results in a large relative change. (**B**) As in A for the FH Ribo-Zero dataset.

Based on the good correlation of all sequencing data (Figure 1B, C and Figure S1B, C) we pooled all RiboMinus tags obtained from each tissue and used this combined data for further analyses. To investigate if the ribo-depleted template preparation method used here was comparable with published polyA RNA-Seq methods, we used RPKM saturation curves [20] to analyse if our sequencing depth of 21 million unique tags (CCE, RiboMinus) and 48 million unique tags (FH, Ribo-Zero) allowed a reliable calculation of gene expression levels for the protein-coding and non-coding part of the RefSeq gene database. Figure 3A shows that with increasing tag number more and more protein-coding genes (left) reach a RPKM value that does not change with further increasing tag number. Importantly, at the final number of obtained tags all genes with an RPKM greater than 3 reached saturation. This indicates that despite the relatively poor ribosomal depletion obtained using the RiboMinus Kit (Figure 1A), we have exhaustively sequenced the protein-coding transcriptome. The picture is similar but delayed for RefSeq ncRNAs as 90% of transcripts with an RPKM greater than 3 have reached saturation at the final number of tags (Figure 3A right). The Ribo-Zero dataset shows a similar picture for protein-coding genes (Figure 3B left) but in this case lowly expressed RefSeq ncRNAs also reach the saturation point (Figure 3B right). This data shows that two different ribosomal RNA depletion approaches produce reliable gene expression data in a similar manner as shown for polyA enriched RNA-Seq [20].

### Ribo-depleted RNA-Seq detects biologically relevant expression differences in protein coding and non-coding RNAs

**Figure 4:**
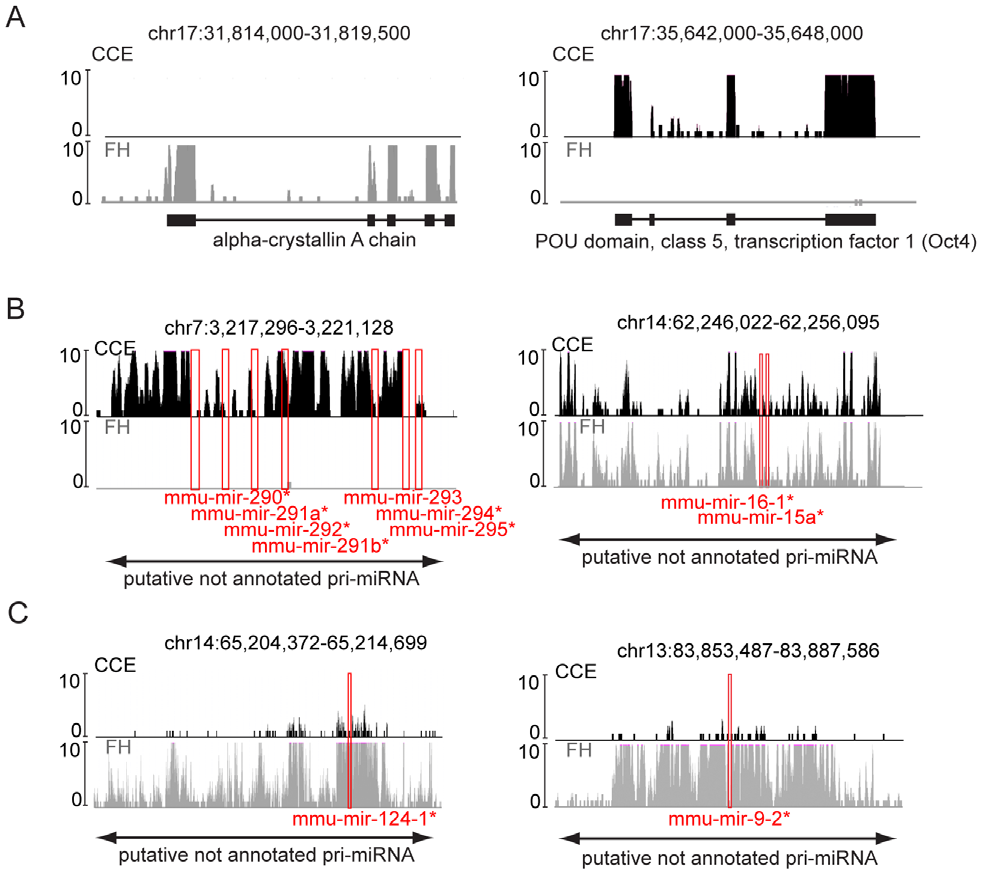
Ribo-depleted RNA-Seq detects tissue specific expression of known protein-coding and non-coding genes. (**A**) UCSC genome browser (http://genome.ucsc.edu/, mm9) screen shots of RNA-Seq data for CCE (black, top) and FH (grey, bottom). The genome position is given on top, black or grey bars indicate the number of sequence tags (tag numbers >10 are cut off) at this position. The position of RefSeq genes (black line) with exons (black boxes), are shown below with the gene name. Left: the *alpha-crystallin A chain* gene is specific for the mouse eye and therefore sequence tags over exons (indicating gene expression) are only found in FH and are absent from CCE. Right: the well-known stem cell marker *Pou5f1* (*Oct4*) shows sequence tags over exons only in CCE but not in FH. (**B**) Putative pri-miRNAs indicating either the specific expression of all miRNAs of the cluster in CCE and the >10 fold reduced expression in FH (left) or the similar expression of the two miRNAs in the region in CCE and FH (right). Red boxes indicate the position of annotated miRNAs (http://genome.ucsc.edu/, mm9) with the name given below. Asterisks mark miRNAs overlapped by a low number of sequence tags. The position of the putative pri-miRNA, not annotated in the RefSeq database, is shown at the bottom by a double-headed arrow. Details as in (A). (**C**) Putative pri-miRNAs indicating the >10 fold increased expression of the single miRNAs of the respective cluster in FH compared to CCE. Details as in (A). Note that in A, B, and C RiboMinus data is shown and that the Ribo-Zero data produced similar results (data not shown).

In the next step we investigated if our ribo-depleted RNA-Seq protocol was useful to detect well-known biological differences in CCE and FH at the level of single genes. For this analysis we first investigated known marker genes for each tissue type in both the RiboMinus and the Ribo-Zero datasets. We found expression of the *alpha-crystallin A chain* gene only in FH, which is specific for the developing eye and therefore absent from CCE cells (Figure 4A left panel). CCE mimics an early embryonic stage [23] and therefore we found the well-known stem cell marker *Pou5f1* (*Oct4*), still expressed in CCE and absent in FH (Figure 4A right panel). We also investigated differentially-expressed genes in both cell types at a global level using the Ingenuity Pathway Analysis (IPA) software for the RiboMinus dataset [30]. This analysis indicates that the top ranked biological processes identify the unique features of both tissues (data not shown).

Tissue specific expression of ncRNAs is less well described in the literature and bioinformatic tools to analyse these genes in a similar way to IPA analysis [30] are not available. However, specific expression of several miRNAs in differentiated ES compared to several mouse tissues has been reported [31]. We could confirm this expression pattern for *mmu-mir-292* and *mmu-mir-293* which are part of a larger pri-miRNA transcript detected exclusively in CCE cells (Figure 4B left panel). Another miRNA, *mmu-mir-16-1*, was reported to be present in differentiated ES cells and several other mouse tissues [31]. In agreement with this finding we identified this miRNA in CCE and FH as a large pri-miRNA transcript (Figure 4B right panel). Mouse eye-specific miRNAs have also been reported and we could confirm increased expression of two of these miRNAs, *mmu-mir-124-1* and *mmu-mir-9-2* in FH, which contains the developing eye (Figure 4C) [32]. Again these miRNAs are part of larger pri-miRNA transcripts.

These examples clearly illustrate that our ribo-depleted RNA-Seq approach not only identifies miRNA precursor transcripts in a similar way to polyA RNA-Seq [20] but also correctly identifies ncRNA expression differences between CCE and FH samples. Together this confirms that this ribo-depleted RNA-Seq protocol is useful for studying the protein-coding and non-coding transcriptome to a level that can be utilised for comparing different datasets.

### Ribo-depleted RNA-Seq is advantageous for detecting macro ncRNAs

**Figure 5:**
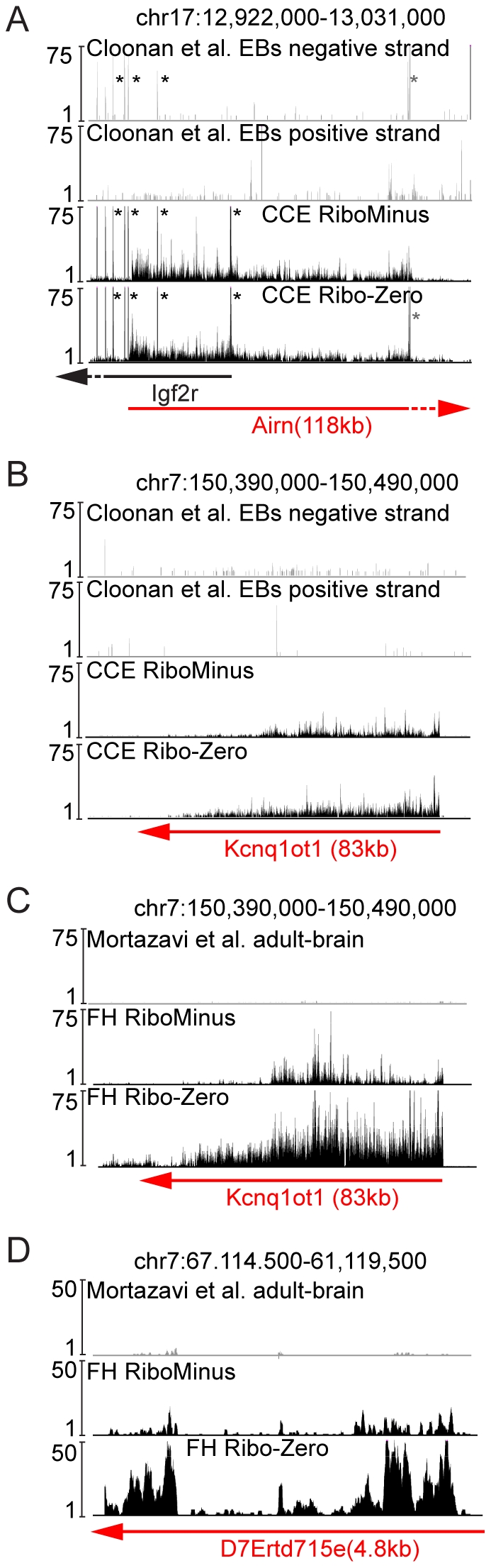
Ribo-depleted RNA-Seq detects macro ncRNAs more efficiently than polyA RNA-Seq. (**A**) UCSC genome browser screen shot as in Figure 4A of the *Airn* macro ncRNA gene. The Cloonan et al. EB polyA RNA-Seq (grey, top), the RiboMinus CCE and Ribo-Zero CCE RNA-Seq data (black, bottom) are shown. Black asterisks mark the signals from the protein-coding mRNA *Igf2r* and grey asterisks mark the position of a pseudogene expressed from chr.15 [41]. Note that the ncRNA *Airn* is 118 kb in length (red arrow, extends outside the region shown) and overlaps exons 2 and 1 protein-coding *Igf2r* gene (black arrow, extends outside the region shown) in antisense orientation. Therefore *Airn* and *Igf2r* signals are visible in the CCE data that has no strand-specific information. For Cloonan et al., strand-specific information was available and *Igf2r* signals are visible on the negative strand (black asterisks, top) whereas only a low amount of signals are visible on the positive strand expressing *Airn*. (**B**) As in A showing the functional 83 kb *Kcnq1ot1* macro ncRNA (red arrow). (**C**) As in B showing Mortazavi et al. adult mouse brain polyA RNA-Seq (grey, top), the RiboMinus FH (black, middle) and Ribo-Zero FH RNA-Seq data (black, bottom). (**D**) As in C showing an annotated RefSeq ncRNA of unknown function. Signals higher than indicated by the scale on the x-axis were cut off. Note that the differences in the read numbers between RiboMinus FH and Ribo-Zero FH reflect the increased number of uniquely aligned tags (see Table S1).

Macro ncRNAs like *Airn* and *Kcnq1ot1* are well-known functional ncRNAs but little is known about their detection by RNA-Seq. Therefore we investigated the ribo-depleted RNA-Seq datasets produced here as well as published ribo-depleted and polyA datasets for the detection of *Airn* and *Kcnq1ot1*. The first published dataset used contains the polyA transcriptome obtained by a SOLiD platform of embryoid body (EB) differentiated ES cells (that have a similar biological origin to the CCE dataset described here, Cloonan et al. [19]). The second dataset used contains polyA selected transcriptome of adult mouse brain (Mortazavi et al. [20]) and was the closest available match to our FH sample for two reasons. First FH is predominantly fetal brain and second this study used a similar template preparation protocol and the same Illumina/Solexa sequencing platform. We first investigated the CCE and Cloonan et al. EB datasets for the expression of the *Airn* and *Kcnq1ot1* macro ncRNAs, which are expressed in both cell types [33]. In both ribo-depleted CCE datasets generated here *Airn* shows continuous expression over its 118 kb long gene body that is absent in the Cloonan et al. EB dataset (Figure 5A). In contrast, the *Igf2r* protein-coding gene was similarly detected in all data sets. The 83 kb long *Kcnq1ot1* macro ncRNAs was similarly not detectable in the Cloonan et al. EB dataset but was present as a continuous region covered with sequencing tags in both ribo-depleted CCE datasets (Figure 5B). *Kcnq1ot1* is widely expressed in the mouse brain [34], however, it was not present in the Mortazavi et al. adult brain datasets but was detectable in both ribo-depleted FH datasets (Figure 5C, Table 1). Interestingly the visual inspection of both *Airn* and *Kcnq1ot1* showed a similar loss of sequence tags towards the 3′ end as shown for long genes. Note that both *Airn* and *Kcnq1ot1* represent a single continuous transcript as the truncation of both macro ncRNAs removed all downstream transcripts [8], [9].

**Table 1:** Macro ncRNAs are more efficiently detected in RiboMinus and Ribo-Zero RNA-Seq.

RefSeq name, region	RPKM expression values				
UCSC genome browser mm9	Mortazavi et al. adult brain polyA [20]	Cui et al. adult brain polyA [22]	Cui et al. adult brain RiboMinus [22]	FH-Ribo-Zero (this study)	FH RiboMinus (this study)
**Airn (NR_027784)** Chr17:12934176–13008423	**0.27**	**0**	**0.5**	**5.7**	**3.33**
**Kcnq1ot1 (NR_001461)** chr7:150399016–150482452	**0.03**	**0.44**	**1.62**	**5.31**	**3.31**
Dlx6os1 (NR_015388)* chr6:6770546–6819533	0.73	7.17	3.76	9.26	9.22
IPW (NR_015351)* chr7:66874528–66934248	0.67	4.57	6.20	5.97	8.19
A330076H08Rik (NR_015599)* chr7:69088787–69127189	0.37	5.78	3.39	6.82	3.69
D7Ertd715e (NR_015456)* chr7:67114463–67119317	0.43	3.80	5.94	8.52	5.26
Ftx (NR_028380)* chrX:100764844–100819093	0.52	13.63	3.78	3.05	3.75

Different ncRNAs annotated in the RefSeq database (http://genome.ucsc.edu/, mm9, RefSeq identifier given in brackets) are shown with their respective RPKM values in two polyA and one RiboMinus RNA-Seq dataset from adult brain (Mortazavi et al., Cui et al., details see text) and the Ribo-Zero as well as the RiboMinus FH datasets presented here. Note that the ncRNAs with unknown function (asterisks) show a reliable RPKM level (>3) in the Cui et al. polyA RNA-Seq datasets and in all the ribosomal RNA depleted datasets. The functional *Airn* and *Kcnq1ot1* macro ncRNAs (bold) that are expressed in mouse brain are only detected in the FH datasets (RPKM>3, details see text).

These results are noteworthy as both *Airn* and *Kcnq1ot1* were reported to be polyadenylated and to contain A-rich regions that could be captured during polyA enrichment [35], [36], [37]. To test if other ncRNAs are preferentially detected in our ribo-depleted RNA-Seq we extended this analysis to other examples of annotated ncRNAs that are expressed in the mouse brain according to the “Affymetrix Exon Array 1.0: Normal Tissues track” on the UCSC genome browser (http://genome.ucsc.edu/, mm9). The two ribo-depleted FH datasets generated here, detected five candidate ncRNAs (RPKM>3) that were not detectable to a reliable level in the Mortazavi et al. adult brain dataset (RPKM<3, *Dlx6os*, *IPW*, *A330076H08Rik*, *D7Ertd715e*, *Ftx* Table 1 and Figure 5D). To further investigate if these ncRNAs were not detectable due to a lack of A-rich regions we investigated their expression in another polyA RNA-Seq dataset, Cui et al. adult mouse brain [22]. Cui et al. used a similar polyA enrichment strategy as Mortazavi et al., but used an enzymatic digestion to fragment the RNA and a SOLiD sequencing platform. The Cui et al. polyA dataset failed to detect the *Airn* and *Kcnq1ot1* macro ncRNAs (RPKM<3, Table 1) but reliably detected all other ncRNAs listed in Table 1 (RPKM>3). Cui et al. also produced a ribo-depleted RNA-Seq dataset using the RiboMinus ribosomal RNA depletion approach and the same fragmentation method and sequencing platform as for the polyA dataset [22]. Interestingly the Cui et al. ribo-depleted dataset also failed to detect the *Airn* and *Kcnq1ot1* macro ncRNAs (RPKM<3, Table 1) but detected all other ncRNAs shown in Table 1 (RPKM>3).

These findings indicate that different polyA RNA-Seq approaches and to a lesser extent, different ribo-depleted RNA-Seq approaches vary in their ability to detect annotated ncRNAs. Importantly the ribo-depleted RNA-Seq template protocol presented here, is advantageous over other tested protocols in the detection of macro ncRNAs, as it is the only one that detected both examples of the functional *Airn* and *Kcnq1ot1* macro ncRNAs. These macro ncRNAs are largely unspliced RNA Polymerase II transcripts with a high proportion of interspersed repeats that have a very short halflife [35]. It is possible that these features make them a difficult target for RNA-Seq and that the advantages of the template preparation protocol described here are the rapid processing of the tissue together with an enzyme-free fragmentation step.

### Ribosomal RNA depletion methods determine the comparability of RNA-Seq datasets

**Figure 6:**
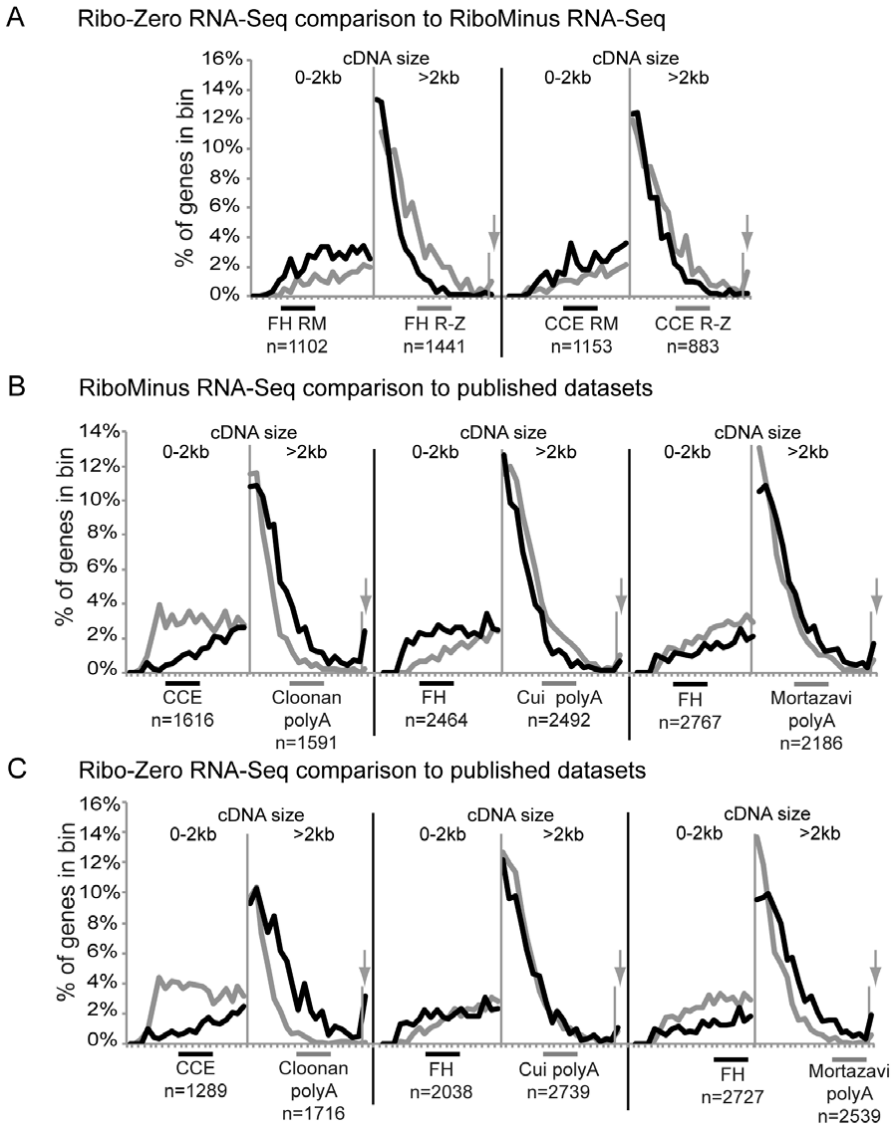
The template preparation protocol determines the comparability of ribo-depleted RNA-Seq to polyA RNA-Seq. The cDNA size distribution of genes showing more than 8× expression difference (Figure S2), in the comparison of (**A**) FH RiboMinus - FH-RiboZero (left) and CCE RiboMinus - CCE Ribo-Zero (right). (**B**) as in A for the comparisons of CCE RiboMinus-Cloonan et al. EB (left), FH RiboMinus-Cui et al. adult mouse brain polyA (middle) and FH RiboMinus-Mortazavi et al. adult mouse brain polyA (right). (**C**) as in A for the comparisons of CCE Ribo-Zero-Cloonan et al. EB (left), FH Ribo-Zero-Cui et al. adult mouse brain polyA (middle) and FH Ribo-Zero-Mortazavi et al. adult mouse brain polyA (right). For Cloonan et al. EB both the gene expression data from the published alignment (shown in B, C, see Materials and methods) and from an alignment done with the pipeline used here (data not shown) were used and produced the same highly significant differences. Two different size classes are shown with different bin sizes (0–2 kb, 100 bp bins and >2 kb, 500 bp bins). Genes bigger than 11.5 kb are grouped in the last bin (arrow).

In the final step of our study we sought to investigate the potential biases of the different ribosomal RNA depletion methods (RiboMinus and Ribo-Zero) presented here in comparison to other RNA-Seq datasets. As no publicly available datasets directly match our datasets we could not compare gene expression levels directly. Therefore we chose to investigate the cDNA length distribution of differentially expressed genes between two datasets, as the length bias is a well established phenomenon distinguishing different template preparation protocols independently of biological differences [17]. We first analysed the length distribution of genes that show differential expression in the RiboMinus compared to the Ribo-Zero datasets (Figure 6A). We found that the RiboMinus datasets detected small genes (<2 kb) significantly more reliably than Ribo-Zero for both FH and CCE (Wilcoxon p<0.05). There was no significant difference between the two ribosomal RNA depletion methods for the detection of large genes (>2 kb, Wilcoxon p>0.05). This result fits with the above observation that small genes are less well covered with sequence tags in the Ribo-Zero sample compared to the RiboMinus sample, which might lead to a less reliable gene expression level in the Ribo-Zero samples (Figure 2B).

We next tested the influence of the RiboMinus and the Ribo-Zero method on the comparability of our datasets to the published datasets introduced above. For determining differentially expressed genes we used a correction factor to maximise the number of genes showing no difference (Figure S2). We found that the Cloonan et al., EB dataset were significantly enriched for small genes (Wilcoxon p< = 0.0006) and depleted for large genes (Wilcoxon p<0.03) compared to the RiboMinus and Ribo-Zero CCE (compare black and grey lines in Figure 6B and C left). In contrast genes differentially expressed in RiboMinus FH sample were significantly enriched for small genes (Wilcoxon p<0.003) but not for large genes (Wilcoxon p>0.05) compared to the Cui et al., adult brain polyA dataset (compare black and grey lines in Figure 6B middle). This difference was not detectable when using the Ribo-Zero FH dataset for the same comparison (Wilcoxon p>0.05, Figure 6C middle). Finally we used the Mortazavi et al. adult brain data and compared it to our FH data. This analysis revealed no difference to the FH RiboMinus dataset for any gene length (compare black and grey lines in Wilcoxon p>0.05, Figure 6B right). When using the FH Ribo-Zero dataset a significant enrichment of small genes (Wilcoxon p<0.003) but not of large genes (Wilcoxon p>0.05) was detectable in the Mortazavi et al. adult brain dataset (Figure 6C right).

It is notable that the difference of the FH and CCE datasets to polyA RNA-Seq datasets that used a column-free RNA preparation protocol (Cui et al. and Mortazavi et al.,) was not as significant (minimum: p<0.003) as the differences to the Cloonan et al. dataset (p<0.0006) that used a column-based RNA preparation method. This indicates that independent of the polyA enrichment or ribosomal RNA depletion, a standardised RNA preparation protocol increases the comparability of the resulting transcriptomes markedly.

### Conclusions

In this study we investigated the use of ribo-depleted RNA-Seq for the study of whole transcriptomes. Using this approach we found differences in the coverage of genes with sequence tags when using different template fragmentation protocols, although not as dramatic as reported previously when comparing studies using different primers for the cDNA synthesis [12]. We show for the first time, that ribo-depleted RNA-Seq produces reliable coding and non-coding gene expression data and is highly reproducible between different sequencing locations and when using different ribosomal RNA depletion strategies. The ribo-depleted RNA-Seq protocol presented here has a clear advantage over comparable published RNA-Seq protocols, as it was the only one to detect the two known functional *Airn* and *Kcnq1ot1* macro ncRNAs. Finally we show that the use of a standardised RNA preparation, improves the comparability of the resulting transcriptome. With this awareness we propose the use of a limited number of tested protocols to allow the sharing of transcriptome data within the scientific community. A ribosomal RNA depletion approach, similar to the one presented here, could be one of these protocols as it is simple but powerful in detecting a large variety of protein-coding and non-protein-coding transcripts. This does not remove the necessity of additional data correction procedures but suggests that a closely matching experimental setup further improves the comparability of the resulting transcriptomes, allowing for even more accurate comparisons after data correction.

## Materials and Methods

### Ethics Statement

No animal experiments according to the Austrian Laboratory Animal Act were performed in this study, because humane killing of laboratory animals is not defined as animal experimentation under the Austrian Laboratory Animal Act (Animal Experiments Act, Federal Law Gazette No. 501/1989). For this reason, approval of the study by an institutional ethics committee was not required. 14.5 days postcoitum mouse embryos were obtained after humane killing of wildtype FVB/N pregnant female mice by cervical dislocation by skilled qualified personnel. Mice were bred and housed at the Forschungsinstitut für Molekulare Pathologie GmbH, Dr. Bohr-Gasse 7, 1030 Vienna, Austria in strict accordance with national recommendations described in the “IMP/IMBA Common Institutional policy concerning the care and use of live animals” with the permission of the national authorities (Laboratory Animal Facility Permit MA58-0375/2007/4).

### RNA preparation

CCE feeder-independent ES cells [38] were grown under standard conditions and differentiation was induced by LIF withdrawal and retinoic acid (0.266 µM final concentration) addition. Mouse fetal head was dissected at 14.5 days postcoitum. Total RNA was isolated from cells and tissues according to a standard TRI reagent (Sigma) protocol and treated with DNase I (Ambion). Tissues were snap frozen in liquid nitrogen before further processing or the Tri reagent was added directly to the cells. 10 µg (Invitrogen RiboMinus™ Transcriptome Isolation Kit Human/Mouse) or 5 µg (Epicentre Ribo-Zero™ rRNA Removal Kit Human/Mouse/Rat) of total RNA from cells or tissues was subjected to ribosomal RNA depletion according to the manufacturer's protocol. Note that the RNAs used for the RiboMinus and the Ribo-Zero preparations are biological replicates. The quality of the purified RNA was determined using the Agilent 2100 Bioanalyzer with the Agilent RNA 6000 Pico Kit according to the manufacturer's protocol.

### RNA fragmentation

RNA-hydrolysis was done as described [20] for 3.5 minutes (RiboMinus) or 4 minutes (Ribo-Zero) and the fragmented RNA was purified with the RNeasy Mini Kit (Qiagen). RNA size and concentration was quantified with the Agilent 2100 Bioanalyzer with Agilent RNA 6000 Pico Kit according to the manufacturer's protocol.

### cDNA preparation

RNA fragmented by hydrolysis or intact RNA was used as template for cDNA synthesis using 5 µg random hexamers (Invitrogen) in a total volume of 20 µl according to the Superscript II Reverse Transcriptase (Invitrogen) standard protocol. Second-strand synthesis was performed by adding 91.8 µl water, 30 µl 5× Second strand buffer (Invitrogen), 3 µl 10 mM dNTP (Invitrogen), 4 µl DNA polymerase I (10 U/µl Invitrogen), 1 µl E. coli DNA ligase (10 U/µl Invitrogen) and 0.2 µl RNase H (2 U/µl Invitrogen), followed by incubation at 16°C for 2 hours. T4 DNA Polymerase (5 U/µl Invitrogen) (1 µl) was added followed by an additional 10 min at 16°C. The ds-cDNA was purified by using the MinElute Reaction Cleanup Kit (Qiagen), according to the manufacturer's protocol.

### cDNA fragmentation

ds-cDNA prepared from intact RNA was fragmented in a volume of 130 µl by a Covaris S1 device according to the manufacturer's protocol using the following conditions: Duty cycle - 15.2%, Intensity - 6.0, Cycles/burst - 500, Duration - 60 sec, Cycle repeat - 5. After ethanol precipitation, the sheared cDNA was recovered in nuclease-free distilled H_2_O.

### Next Generation Sequencing

Vienna-IMP and Nijmegen sequencing locations performed these steps independently using the same machines and the same ds-cDNA starting material. NGS in Nijmegen was done as described for libraries with a fragment size of 300 bp [39]. In Vienna-IMP 10 ng of fragmented cDNA was used to generate sequencing libraries with a fragment size of 200–700 bp as described by Illumina's ChIP-Seq sample preparation protocol. Libraries were quantified with the Agilent Bioanalyzer dsDNA 1000 assay Kit. Cluster generation and single read sequencing was carried out using Illumina/Solexa Genome Analyzer (GA) II systems according to the manufacturer's guidelines with a 36 bp read length for all samples. In Vienna-CeMM the procedure was essentially the same with the exception that 5 ng of cDNA was used to produce sequencing libraries with a fragment size of 150–600 bp. Cluster generation and single read sequencing was carried out using Illumina/Solexa HiSeq 2000 systems according to the manufacturer's guidelines with a 51 bp read length for all samples. All sequencing data has been submitted to the GEO database: Accession number GSE22959.

### Data analysis

High quality tags were aligned against mitochondrial sequences (mm9, http://genome.ucsc.edu/, RiboMinus samples) and rRNA sequences (GenBank BK000964.1, RiboMinus and Ribo-Zero samples). Remaining tags were aligned to the mouse genome (mm9, http://genome.ucsc.edu/) using BOWTIE [40], not considering individual base pair qualities and allowing 2 mismatches for 36 bp tags and 3 mismatches for 51 bp tags. Unique tags were defined as matching only once in the genome and tags with more than one match were flagged as repeats and excluded in subsequent analyses. We did not map tags covering splice junctions as a previous study found that less than 1% of tags map to exon-exon junctions in RiboMinus RNA-Seq [22].

### Databases

The RefSeq (mm9, http://genome.ucsc.edu/, freeze: 2009-07-24) entries were partitioned into a non-coding and protein-coding fraction: 1. non-coding RNA: NCBI was searched using “ncRNA RNA” AND “Mus musculus” 2. Protein-coding: all RefSeq entries occurring in the non-coding RNA list and all entries that start with “NR_” were removed. The resulting list was the RefSeq protein-coding gene set.

### RPKM/PPKM

RPKM (Reads Per Kilobase of exon model per Million of reads) was calculated as described [20] for the exons of the transcripts in the respective RefSeq database. The pileup defines the number of sequence tags covering each base of the genome. The PPKM (Pileup Per Kilobase of exon model per Millions of reads) is calculated as the RPKM but instead of the actual tags, the sum of the pileups of each base in the respective exons was used. RPKM and PPKM are highly correlated and a factor of 30 can be used to convert RPKM to PPKM (RPKM 3 = PPKM 90).

### Scatter plots

For scatter plots and Pearson correlation RPKM values were adjusted +1 and log10 transformed.

### Analysis of published datasets

Detailed information about the aligned sequence tags (pileups) was available from the Cloonan et al. dataset [19] and was used to calculate expression values (PPKM - Pileup Per Kb per Millions of tags). This data was not available for the Cui et al. and the Mortazavi et al. adult brain dataset [22], so we realigned the publicly available sequence tags and used this to obtain RPKM expression values.

### Statistical analysis

t-tests were done in EXCEL: 1 tail, two-sample equal variance. For the comparison of the different tag types the percentages of tags were compared among all relevant, available replicates from the list shown in Table S1. The Wilcoxon Two Sample Test was calculated at http://www.fon.hum.uva.nl/Service/Statistics/Wilcoxon_Test.html.

### Gene coverage (5′-3′ bias)

All RefSeq protein-coding genes that were expressed in the CCE and FH samples at an RPKM>3, were divided into 100 bins and the number of tags in each bin was determined separately for all sheared and all hydrolysed RiboMinus datasets and for the FH as well as the CCE Ribo-Zero datasets and normalised for the tag size and the total number of tags. Protein-coding genes were analysed as coding exons and UTRs separate, based on the RefSeq information. For the display UTRs bins were combined (averaged) into 10 bins. The bins were plotted relative to the bin with the highest tag count.

### Size distribution

The expression ratios of the RPKM/PPKM values (corrected to maximize the number of genes with expression differences smaller than 8×, see Figure S2) for RefSeq protein-coding genes were calculated. Genes with an RPKM<3 were considered to be not expressed. Differential expression was defined as a >8× expression difference. The genes in each set were grouped into bins differing by 100 bp in size if the cDNA was shorter than 2 kb or in bins differing by 500 bp in size if the cDNA was bigger than 2 kb using cDNA sizes defined by RefSeq. cDNAs larger than 11.5 kb were grouped into the last bin. The amount of genes in each bin was plotted relative to the total amount of genes in the set. For the statistical analyses the relative gene number was used.

## Supporting Information

Figure S1 **Optimisation and reproducibility of ribo-minus RNA-Seq in 14.5 dpc fetal head (FH).** (**A**) Similar analysis as in Figure 1A for sequence tags obtained from FH tissue. Samples were depleted for ribosomal RNAs once (lanes nr.1,2,4,5,7), or twice (lane nr. 3 and 6) using RiboMinus and once using Ribo-Zero (lane nr.8). Note that the tag composition of lane nr.2 differs from all other RiboMinus samples and therefore most likely is a technical outlier. (**B**) Similar analysis as in Figure 1B for sequence tags obtained from FH tissue. (**C**) Similar analysis as in Figure 1C for sequence tags obtained from FH tissue.
(PDF)

Figure S2 **Determining correction factors for the calculation of differentially expressed genes.** The number of genes showing expression differences as indicated by the bins on the x-axis was calculated for RefSeq protein-coding genes for the following RiboMinus (RM) datasets (shown in Figure 6B): (**A**) FH-RM – Cui et al. polyA, (**B**) CCE-RM – Cloonan et al. EBs (**C**) Mortazavi et al. adult brain - FH-RM (first tissue is shown on the left, second tissue is shown on the right). Note that the 0 bin contains all genes showing no expression in both datasets. Expression is defined by an expression value larger than RPKM 3 for all comparisons except for CCE-RM - Cloonan et al. EB, where a cutoff of PPKM 90 was used. All comparisons were corrected to maximize the number of genes in the bins with expression differences smaller than 8× except for (A) where no correction was necessary. Differential expression was defined as a larger than 8 fold expression difference. Grey bars show the number of genes before the correction, black bars show the number of genes after the correction. Note that the correction factor is shown in the figure and that the expression values for the tissue shown right were multiplied with this factor. For the Ribo-Zero comparisons (shown in Figure 6C) the same analysis was performed (data not shown) and the following correction factors were used (RPKM values of the dataset written left were multiplied with this factor): Cloonan et al. EB - CCE-RZ: 3.5, FH-RZ - Cui et al. polyA: no correction, FH-RZ - Mortazavi et al. adult brain: 1.5.
(PDF)

Table S1 **Ribosome depletion, tag types and GEO accession numbers obtained in the individual sequencing reactions.**
(DOC)

## References

[1] Carninci P, Kasukawa T, Katayama S, Gough J, Frith MC (2005). The transcriptional landscape of the mammalian genome.. Science.

[2] Kapranov P, Cawley SE, Drenkow J, Bekiranov S, Strausberg RL (2002). Large-scale transcriptional activity in chromosomes 21 and 22.. Science.

[3] Katayama S, Tomaru Y, Kasukawa T, Waki K, Nakanishi M (2005). Antisense transcription in the mammalian transcriptome.. Science.

[4] Mattick JS, Taft RJ, Faulkner GJ (2010). A global view of genomic information–moving beyond the gene and the master regulator.. Trends in genetics : TIG.

[5] Zamore PD (2010). Somatic piRNA biogenesis.. EMBO J.

[6] Kim VN, Han J, Siomi MC (2009). Biogenesis of small RNAs in animals.. Nat Rev Mol Cell Biol.

[7] Matera AG, Terns RM, Terns MP (2007). Non-coding RNAs: lessons from the small nuclear and small nucleolar RNAs.. Nat Rev Mol Cell Biol.

[8] Mancini-Dinardo D, Steele SJ, Levorse JM, Ingram RS, Tilghman SM (2006). Elongation of the Kcnq1ot1 transcript is required for genomic imprinting of neighboring genes.. Genes Dev.

[9] Sleutels F, Zwart R, Barlow DP (2002). The non-coding Air RNA is required for silencing autosomal imprinted genes.. Nature.

[10] Williamson CM, Ball ST, Dawson C, Mehta S, Beechey CV (2011). Uncoupling antisense-mediated silencing and DNA methylation in the imprinted Gnas cluster.. PLoS genetics.

[11] Wilhelm BT, Landry JR (2009). RNA-Seq-quantitative measurement of expression through massively parallel RNA-sequencing.. Methods.

[12] Wang Z, Gerstein M, Snyder M (2009). RNA-Seq: a revolutionary tool for transcriptomics.. Nat Rev Genet.

[13] Metzker ML (2010). Sequencing technologies - the next generation.. Nat Rev Genet.

[14] Pepke S, Wold B, Mortazavi A (2009). Computation for ChIP-seq and RNA-seq studies.. Nat Methods.

[15] Costa V, Angelini C, De Feis I, Ciccodicola A (2010). Uncovering the complexity of transcriptomes with RNA-Seq.. J Biomed Biotechnol.

[16] Roberts A, Trapnell C, Donaghey J, Rinn JL, Pachter L (2011). Improving RNA-Seq expression estimates by correcting for fragment bias.. Genome Biol.

[17] Oshlack A, Wakefield MJ (2009). Transcript length bias in RNA-seq data confounds systems biology.. Biol Direct.

[18] Shi L, Reid LH, Jones WD, Shippy R, Warrington JA (2006). The MicroArray Quality Control (MAQC) project shows inter- and intraplatform reproducibility of gene expression measurements.. Nat Biotechnol.

[19] Cloonan N, Forrest AR, Kolle G, Gardiner BB, Faulkner GJ (2008). Stem cell transcriptome profiling via massive-scale mRNA sequencing.. Nat Methods.

[20] Mortazavi A, Williams BA, McCue K, Schaeffer L, Wold B (2008). Mapping and quantifying mammalian transcriptomes by RNA-Seq.. Nat Methods.

[21] Ramskold D, Wang ET, Burge CB, Sandberg R (2009). An abundance of ubiquitously expressed genes revealed by tissue transcriptome sequence data.. PLoS Comput Biol.

[22] Cui P, Lin Q, Ding F, Xin C, Gong W (2010). A comparison between ribo-minus RNA-sequencing and polyA-selected RNA-sequencing.. Genomics.

[23] Chen AC, Gudas LJ (1996). An analysis of retinoic acid-induced gene expression and metabolism in AB1 embryonic stem cells.. J Biol Chem.

[24] t Hoen PA, Ariyurek Y, Thygesen HH, Vreugdenhil E, Vossen RH (2008). Deep sequencing-based expression analysis shows major advances in robustness, resolution and inter-lab portability over five microarray platforms.. Nucleic Acids Res.

[25] Armour CD, Castle JC, Chen R, Babak T, Loerch P (2009). Digital transcriptome profiling using selective hexamer priming for cDNA synthesis.. Nature methods.

[26] Castle JC, Armour CD, Lower M, Haynor D, Biery M (2010). Digital genome-wide ncRNA expression, including SnoRNAs, across 11 human tissues using polyA-neutral amplification.. PloS one.

[27] Nagalakshmi U, Wang Z, Waern K, Shou C, Raha D (2008). The transcriptional landscape of the yeast genome defined by RNA sequencing.. Science.

[28] Pesole G, Mignone F, Gissi C, Grillo G, Licciulli F (2001). Structural and functional features of eukaryotic mRNA untranslated regions.. Gene.

[29] Schwartz S, Oren R, Ast G (2011). Detection and removal of biases in the analysis of next-generation sequencing reads.. PLoS One.

[30] Xu G, Fewell C, Taylor C, Deng N, Hedges D (2010). Transcriptome and targetome analysis in MIR155 expressing cells using RNA-seq.. RNA.

[31] Houbaviy HB, Murray MF, Sharp PA (2003). Embryonic stem cell-specific MicroRNAs.. Dev Cell.

[32] Karali M, Peluso I, Marigo V, Banfi S (2007). Identification and characterization of microRNAs expressed in the mouse eye.. Invest Ophthalmol Vis Sci.

[33] Latos PA, Stricker SH, Steenpass L, Pauler FM, Huang R (2009). An in vitro ES cell imprinting model shows that imprinted expression of the Igf2r gene arises from an allele-specific expression bias.. Development.

[34] Mercer TR, Dinger ME, Sunkin SM, Mehler MF, Mattick JS (2008). Specific expression of long noncoding RNAs in the mouse brain.. Proc Natl Acad Sci U S A.

[35] Koerner MV, Pauler FM, Huang R, Barlow DP (2009). The function of non-coding RNAs in genomic imprinting.. Development.

[36] Furuno M, Pang KC, Ninomiya N, Fukuda S, Frith MC (2006). Clusters of internally primed transcripts reveal novel long noncoding RNAs.. PLoS Genet.

[37] Pandey RR, Mondal T, Mohammad F, Enroth S, Redrup L (2008). Kcnq1ot1 antisense noncoding RNA mediates lineage-specific transcriptional silencing through chromatin-level regulation.. Mol Cell.

[38] Pauler FM, Stricker SH, Warczok KE, Barlow DP (2005). Long-range DNase I hypersensitivity mapping reveals the imprinted Igf2r and Air promoters share cis-regulatory elements.. Genome Res.

[39] Marks H, Chow JC, Denissov S, Francoijs KJ, Brockdorff N (2009). High-resolution analysis of epigenetic changes associated with X inactivation.. Genome Res.

[40] Langmead B, Trapnell C, Pop M, Salzberg SL (2009). Ultrafast and memory-efficient alignment of short DNA sequences to the human genome.. Genome Biol.

[41] Lyle R, Watanabe D, te Vruchte D, Lerchner W, Smrzka OW (2000). The imprinted antisense RNA at the Igf2r locus overlaps but does not imprint Mas1.. Nat Genet.

