# Supplementary figures and images for: An RNA-Seq Strategy to Detect the Complete Coding and Non-Coding Transcriptome Including Full-Length Imprinted Macro ncRNAs
Source: PLoS One. 2011 Nov 10;6(11):e27288. doi: 10.1371/journal.pone.0027288 (PMC3213133; doi:10.1371/journal.pone.0027288)

Figure S1

A

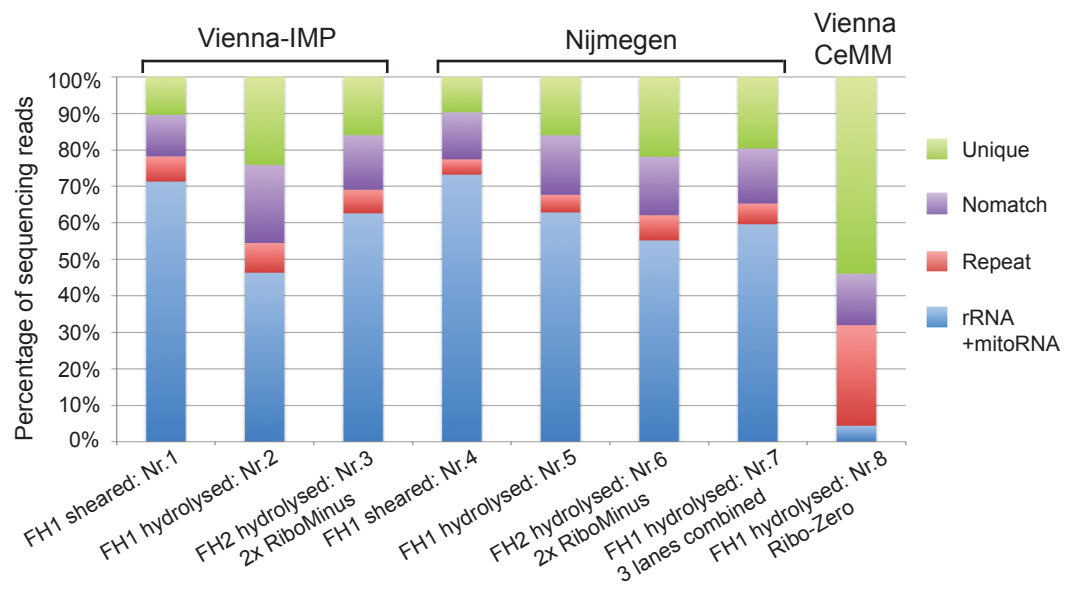

B

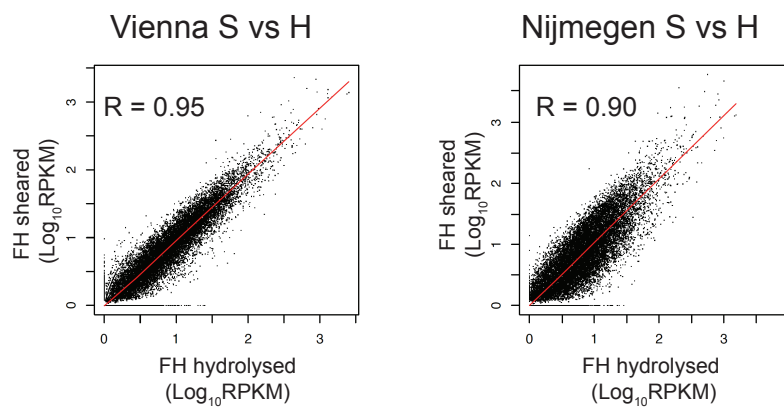

C

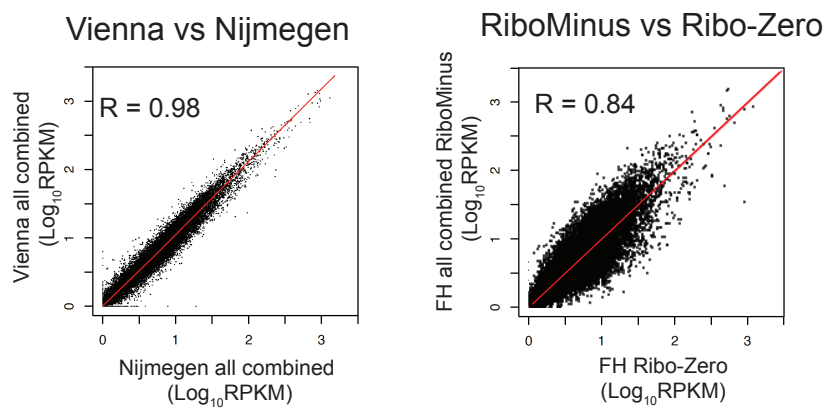

Supplement: Figure S1 — Optimisation and reproducibility of ribo-minus RNA-Seq in 14.5 dpc fetal head (FH). (A) Similar analysis as in Figure 1A for sequence tags obtained from FH tissue. Samples were depleted for ribosomal RNAs once (lanes nr.1,2,4,5,7), or twice (lane nr. 3 and 6) using RiboMinus and once using Ribo-Zero (lane nr.8). Note that the tag composition of lane nr.2 differs from all other RiboMinus samples and therefore most likely is a technical outlier. (B) Similar analysis as in Figure 1B for sequence tags obtained from FH tissue. (C) Similar analysis as in Figure 1C for sequence tags obtained from FH tissue. (PDF) [file pone.0027288.s001.pdf]

Figure S2

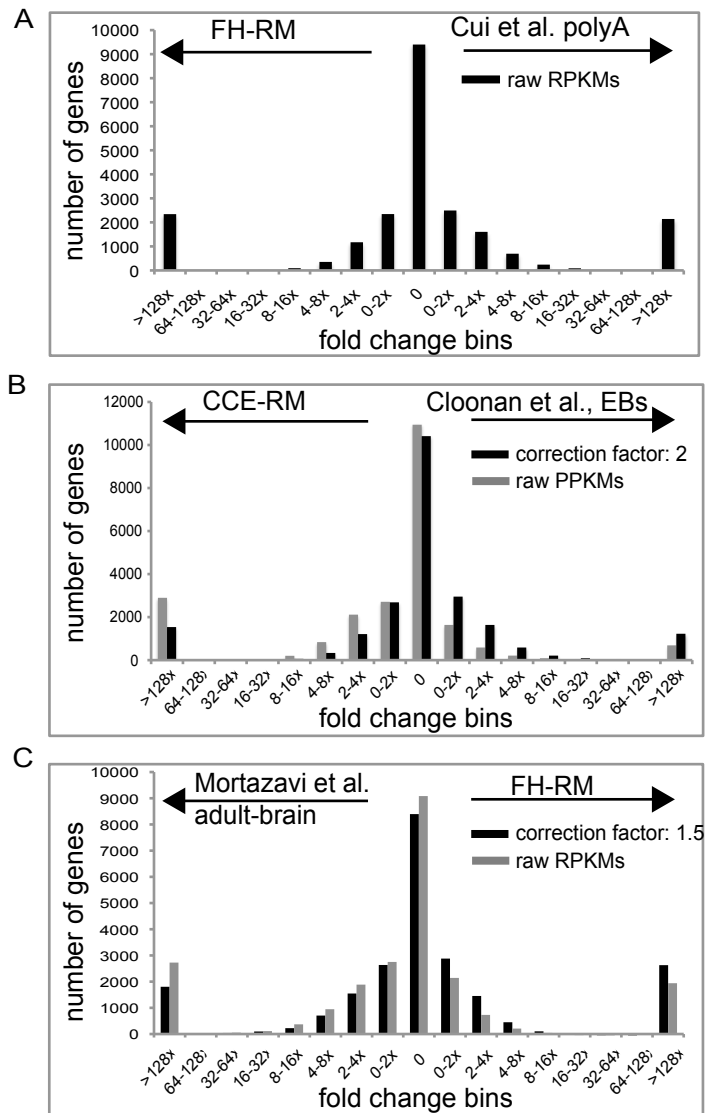

Supplement: Figure S2 — Determining correction factors for the calculation of differentially expressed genes. The number of genes showing expression differences as indicated by the bins on the x-axis was calculated for RefSeq protein-coding genes for the following RiboMinus (RM) datasets (shown in Figure 6B): (A) FH-RM – Cui et al. polyA, (B) CCE-RM – Cloonan et al. EBs (C) Mortazavi et al. adult brain - FH-RM (first tissue is shown on the left, second tissue is shown on the right). Note that the 0 bin contains all genes showing no expression in both datasets. Expression is defined by an expression value larger than RPKM 3 for all comparisons except for CCE-RM - Cloonan et al. EB, where a cutoff of PPKM 90 was used. All comparisons were corrected to maximize the number of genes in the bins with expression differences smaller than 8× except for (A) where no correction was necessary. Differential expression was defined as a larger than 8 fold expression difference. Grey bars show the number of genes before the correction, black bars show the number of genes after the correction. Note that the correction factor is shown in the figure and that the expression values for the tissue shown right were multiplied with this factor. For the Ribo-Zero comparisons (shown in Figure 6C) the same analysis was performed (data not shown) and the following correction factors were used (RPKM values of the dataset written left were multiplied with this factor): Cloonan et al. EB - CCE-RZ: 3.5, FH-RZ - Cui et al. polyA: no correction, FH-RZ - Mortazavi et al. adult brain: 1.5. (PDF) [file pone.0027288.s002.pdf]
