# Supplementary material for: An RNA-Seq Strategy to Detect the Complete Coding and Non-Coding Transcriptome Including Full-Length Imprinted Macro ncRNAs
Source: PLoS One. 2011 Nov 10;6(11):e27288. doi: 10.1371/journal.pone.0027288 (PMC3213133; doi:10.1371/journal.pone.0027288)
Supplement: Table S1 — Ribosome depletion, tag types and GEO accession numbers obtained in the individual sequencing reactions. (DOC) [file pone.0027288.s003.doc]

**Table S1. Ribosome depletion, tag types and GEO accession numbers obtained in the individual sequencing reactions**

| **Lane** | **rRNA depletion** | **Total reads** | **Uniquereads** | **No-match reads** | **Repeat reads** | **rRNA+MchrRNA+** | **Sequencing Location** | **GEO Accession No.** |
| --- | --- | --- | --- | --- | --- | --- | --- | --- |
| CCE1 sheared | 1 round | 15593687 | 1928324 | 1415618 | 1321078 | 10928667 | Vienna | GSM566792 |
| CCE2 sheared | 1 round | 9593408 | 1309208 | 1295350 | 669640 | 6319210 | Vienna | GSM566794 |
| CCE1 hydrolyzed | 1 round | 14557115 | 2473448 | 2039391 | 1122180 | 8922096 | Vienna | GSM566793 |
| CCE2 hydrolyzed | 1 round | 9296010 | 1452794 | 1311420 | 820828 | 5710968 | Vienna | GSM566795 |
| 14.5dpc FH1 sheared | 1 round | 9330937 | 968337 | 1062355 | 647217 | 6653028 | Vienna | GSM566796 |
| 14.5dpc FH1 hydrolyzed | 1 round | 8779272 | 2115848 | 1881557 | 714622 | 4067245 | Vienna | GSM566797 |
| 14.5dpc FH2 hydrolyzed | 2 rounds | 13709811 | 2188956 | 2049737 | 888084 | 8583034 | Vienna | GSM566798 |
| CCE1 sheared | 1 round | 9423824 | 1058045 | 1138779 | 653860 | 6573140 | Nijmegen | GSM566799 |
| CCE2 sheared | 1 round | 9411194 | 1395877 | 1355497 | 520568 | 6139252 | Nijmegen | GSM566801 |
| CCE1 hydrolyzed | 1 round | 9818566 | 1518642 | 1424631 | 756722 | 6118571 | Nijmegen | GSM566800 |
| CCE2 hydrolyzed | 1 round | 10239178 | 1913424 | 1521196 | 728585 | 6075973 | Nijmegen | GSM566802 |
| 14.5dpc FH1 sheared | 1 round | 9498998 | 920424 | 1225185 | 399621 | 6953768 | Nijmegen | GSM566803 |
| 14.5dpc FH1 hydrolyzed | 1 round | 10004482 | 1597887 | 1633895 | 484938 | 6287762 | Nijmegen | GSM566804 |
| 14.5dpc FH2 hydrolyzed | 2 rounds | 9760125 | 2133462 | 1565406 | 676620 | 5384637 | Nijmegen | GSM566805 |
| CCE2 hydrolyzed 3 lanes combined | 1 round | 35889263 | 7958909 | 4947235 | 2963075 | 20020044 | Nijmegen | GSM566806 ~ GSM566808 |
| 14.5dpc FH1 hydrolyzed 3 lanes combined | 1 round | 36331485 | 7129104 | 5478525 | 2061938 | 21661918 | Nijmegen | GSM566809 ~ GSM566811 |
| CCE hydrolyzed | RiboZero | 79766249 | 28708715 | 16682652 | 23580536 | 10794346 | CeMM | GSM718982 |
| 14.5dpc FH hydrolyzed | RiboZero | 89895136 | 48426051 | 12675869 | 24770457 | 4022759 | CeMM | GSM718983 |

Abbreviations as in Figure 1. Ribosomal RNAs were removed using the RiboMinus KIT unless indicated otherwise. For the RiboMinus samples no-match, repeat, rRNA and mitochondrial tags were removed for further analysis; for the Ribo-Zero samples no-match, repeat and rRNA tags were removed for further analysis.
